# Efficacy of Peer Education for Adopting Preventive Behaviors against Head Lice Infestation in Female Elementary School Students: A Randomised Controlled Trial

**DOI:** 10.1371/journal.pone.0169361

**Published:** 2017-01-10

**Authors:** Mahdi Moshki, Fereshteh Zamani-Alavijeh, Mehdi Mojadam

**Affiliations:** 1 Public Health Department, School of Health Sciences, Social Development & Health Promotion Research Center, Gonabad University of Medical Sciences, Gonabad, Iran; 2 Health Education and Promotion Department, School of Health, Isfahan University of Medical Sciences, Isfahan, Iran; 3 Public Health Department, School of Health, Jundishapur University of Medical Sciences, Ahvaz, Iran; TNO, NETHERLANDS

## Abstract

**Background:**

Pediculosis is a common parasitic infestation in students worldwide, including Iran. This condition is more prevalent in populous and deprived communities with poor personal hygiene. This study sought to assess the efficacy of peer education for adopting preventive behaviors against pediculosis in female elementary school students based on the Health Belief Model (HBM).

**Methods:**

A total of 179 female fifth grade students were selected using multistage random sampling and were randomly allocated to control and intervention groups. A standard questionnaire was designed and administered to collect baseline information. An educational intervention was then designed based on the conducted needs assessment. The educational program consisted of three sessions, held by peers for the intervention group. The questionnaire was re-administered one month after the intervention. Independent and paired t-test, Pearson’s correlation coefficient, and regression analysis were applied as appropriate.

**Results:**

The two groups had no significant differences in the scores of knowledge, HBM constructs, or behavior before the intervention. After the intervention, however, the mean scores of all parameters significantly improved in the intervention group.

**Conclusion:**

Peer education based on HBM is an effective strategy to promote preventive behaviors against pediculosis in among fifth grade female elementary school students in Iran.

## Introduction

The head louse is a parasitic arthropod with incomplete metamorphosis. Among the three types of human lice, i.e. head, body, and pubic lice [1], head lice were the first identified species in humans [2]. In the United States, 367 million dollars are annually spent to treat and control 6–12 million cases of infestation with head lice [3]. Head lice infestation, known as pediculosis, is widely seen in all parts of the world including Iran. This condition is more prevalent in areas with high population density, high levels of poverty, and poor personal hygiene [4]. Human lice cannot survive long away from their host [5]. They bite their hosts, suck their blood, and inject them with saliva several times a day. The toxins in the lice saliva can cause various symptoms, including fatigue, irritability, pessimism, and lethargy, in the host. Allergic reactions, such as extreme itchiness, are also common among the hosts. Headache, a feeling of heaviness in the limbs, muscle cramps, insomnia, and lack of concentration have also been observed in children with lice [6]. Furthermore, inadequate knowledge of family members, school staff, and health workers about louse infestation may have psychological effects on not only the patients, but also their family members and the society [7].

Head lice are more common among children (aged 3–11 years) and females (who have more hair) [6]. Since head lice can be transmitted through either direct (head-to-head) contact with the infested person or contact with his/her personal items such as comb, hair brush, hat, scarf, bedding, and towels [6,8,9], crowded places, e.g. schools, provide favorable conditions for the spread of lice [10]. According to the American Center for Disease Control and Prevention, prevalence rates over 5% indicate an epidemic of lice infestation [11]. The prevalence of pediculosis in the primary schools of developed countries has been estimated at 2%-10%. A variety of reasons, such as overpopulation, rural-urban migration, suburbanization, and formation of satellite towns with minimum level of welfare and hygiene, have turned pediculosis into a major health hazard (along with other communicable diseases) in different parts of Iran [12]. The prevalence of pediculosis varies from 6% to 30% in different areas of Iran [13] and has been reported to be 9.2% in Amlash (Guilan Province), 1.8% in Kerman, 7.6% in Qom, and 11% in Ahwaz [5,8,12,13] cities. A relatively high prevalence of pediculosis (up to 10.28%) has been reported in Gonabad (Khorasan Razavi Province), especially among the female fifth graders [14,15].

Health education is essential to enhance public knowledge about the preventability of pediculosis [6]. The first step in planning for any health education program would be to choose an appropriate pattern of education. The Health Belief Model (HBM) is one of the oldest models in which the theories of behavioral sciences are used for resolution of health problems, particularly disease prevention. Over the past 50 years, the HBM has been successfully applied to deal with different health problems. This comprehensive model highlights the relation between beliefs and behaviors and argues that preventive behaviors are formed based on personal beliefs about one’s susceptibility to a disease, the effects of the disease on one’s life, and the efficacy of health measures to decrease susceptibility and disease severity [16]. The HBM domains include perceived susceptibility(the subjective belief that one might catch a disease or a health condition due to a specific behavior), perceived severity (the subjective belief about the extent of damage caused by a disease or health problem following a specific behavior), perceived barriers (belief about the potential costs of a new behavior), perceived benefits (beliefs about the advantages of a proposed method for reducing the risk or severity of a disease or the damage caused by a specific behavior), cues to action (an accelerating force that makes a person feel the need for a particular action), and self-efficiency (one’s perception of his/her ability to adopt a new behavior) [17].

Different studies have confirmed the efficacy of the HBM in promoting preventive behaviors. The model has, in fact, been successfully applied to prevent giardiasis [18], promote desirable dietary behaviors in students [19] and prevent urinary tract infections in pregnant women [20].

Peer education is a common approach to encourage health-enhancing behaviors. Trained peers can effectively communicate with their peers and transfer information throughways which cannot be used by the health personnel. Since they may be perceived as tangible role models by their peers, they can evoke feelings of empathy and trust and serve as good links between health centers and schools [21]. Previous studies have reported the usefulness of peer education in formation of good eating habits among students [19], promotion of breast self-examination in university students [21], and health education in adolescents [22].

Despite the high prevalence of pediculosis in elementary school students, especially in females, studies on the efficacy of peer education based on the HBM to prevent pediculosis in this age group are scarce. Therefore, this study aimed to assess the efficacy of peer education using the HBM for adopting preventive behaviors against pediculosis in female elementary school students.

## Materials and Methods

### Design and Measure

This randomised controlled trial was conducted during the 2013 academic year. Upon gaining the approval of the Research Ethics Committee of Gonabad University of Medical Sciences (GMU/RCT/91/10), researchers visited the Ministry of Education, explained the research objectives to the authorities, and obtained the required permissions to perform this study in elementary schools of Gonabad city (in northeast of Iran). The study was conducted in accordance with the Declaration of Helsinki. Participation of students in the study was voluntarily and written informed consent was obtained from the parents of students who were willing to participate in the study.

A list of elementary schools for girls was obtained from the authorities. Using multistage cluster sampling, the city was divided into two districts, and two schools were randomly selected out of 13 schools in each district. Of the two selected schools, one served as the intervention and the other as the control school in each district. The intervention group (n = 87) was randomly recruited among the fifth graders of the intervention school. A total of 92 fifth graders were randomly enrolled from the control schools and served as the control group. We selected fifth graders for this study since previous studies on the prevalence of head lice, particularly in Gonabad, had reported the highest prevalence of head lice in this age group (its prevalence is lower in younger and older students) [14,15,23].

Afterward, a member of our research team visited the schools, described the study objectives, and motivated the students to participate in the study. Female fifth graders were included if they were in the age range of 10–12 years, had attended the same class from the beginning of the educational year, had no lice infestation based on self-report and were willing to participate in the study and the educational course. The exclusion criteria were missing educational sessions, failure to complete the questionnaire, and having lice infestation. No student was included or excluded during the course of study.

Since there were no standard questionnaires in this field, a researcher-made questionnaire was designed based on the existing literature. In order to ensure the content and face validity of the questionnaire, a panel of 10 experts (in health education, medical entomology, infectious diseases, and public health) was asked to express their opinions and comments regarding the questionnaire items. Moreover, five elementary school teachers and 10 eligible students who were not included in the study were asked to read and answer the questions and confirm their clarity. The required modifications were finally made to the questionnaire and its final version was used for the study.

The reliability of the questionnaire was also determined after it was completed by 20 students (other than the participants). The calculated Cronbach’s alpha was 77% for the whole scale and 86%, 82%, 78%, 85%, 74%, 76%, and 78% for the knowledge, perceived susceptibility, perceived severity, perceived barriers, perceived benefits, self-efficiency, and behavior subscales, respectively.

The designed self-administered questionnaire was comprised of four parts. The first part included demographic information such as age, birth order, and parents’ occupation and educational level. The second part contained nine yes/no questions about the students’ level of knowledge about lice, its transmission and prevention. In this part, incorrect and correct answers were scored as zero and two, respectively. The total scores ranged from zero to 18. The third part dealt with the constructs of the HBM including perceived susceptibility, perceived severity, perceived barriers, perceived benefits, and self-efficiency. Each construct was evaluated by five items scored on a five-point Likert scale from one (strongly disagree) to five (strongly agree). It is noteworthy that the scoring was reversed in case of perceived barriers. Items regarding the cues to action were also assessed as frequency and percentage. The fourth part collected data about behaviors to prevent pediculosis. The five items in this part had three-choice answers (always, sometimes, and never) and were scored from zero to two.

The questionnaires were distributed among the intervention and control groups and the completed questionnaires were analyzed to identify the educational needs of students. The educational content was then planned based on the determined needs. Six students who acquired the highest knowledge scores, were willing to act as a peer educator, and had the required potentials such as speech skills (as determined by their teachers) were selected from the intervention group. The selected six peer educators attended a one-day workshop and were trained by the researcher to acquire sufficient skills and knowledge for peer education. A musical puppet show was used to reinforce the educational content and motivate the educators. Finally, the training package was given to peer educators.

In order to sensitize the students, some leaflets on lice prevention were installed in schools, before the education. The peer trainers held the educational course, comprising of three 45-minute sessions, in a classroom where the researcher was also present. In the first session, the students were sensitized to the issue, and the cause and source of infestation (perceived susceptibility and perceived severity) were discussed through lecture and slide presentation. In the second session, the routes of transmission and prevention of this condition were discussed by showing short films and lecture. During this session, the parents and teachers were provided with pamphlets (cues to action). The last session was a question-and-answer session in which the presented topics were reviewed and the benefits and barriers to prevention of pediculosis were discussed. Post-test was conducted one month after termination of the educational course.

### Data analysis

After collecting all the completed questionnaires, data were coded and analyzed using descriptive statistics (e.g. frequency, mean, and standard deviation) and inferential statistics (including chi-square test, independent and paired t-tests, Pearson’s correlation coefficient, and multivariate linear regression analysis). All analyses were performed using SPSS version 20 (SPSS Inc., Chicago, IL, USA) at a significance level of 0.05. Normal distribution of data was determined using the Kolmogorov-Smirnov test.

## Results

Table 1 presents the demographic characteristics of the intervention and control groups. As seen, most students in both groups were 11 years old, first-born children, and from families with four-five members. The mean frequency of bathing was 2.50 ± 1.18 times a week in the intervention group and 2.46 ± 1.01 times a week in the control group. The mean frequency of combing was 2.72 ± 1.80 and 2.60 ± 1.41 times a day in the intervention and control groups, respectively (Table 2). Based on the chi-square test results, the two groups had no significant differences in terms of age, birth order, family size, parents’ occupation, or frequency of bathing and combing before the intervention (P ≥ 0.05).

**Table 1:** Frequency distribution of the students’ demographic information in the intervention and control groups.

Demographics Variables		Intervention group		Control group		P value*
		n	%	n	%	
**Age**						
	**10 years**	10	11.5	3	3.3	0.460
	**11 years**	49	56.3	63	68.5	
	**12 years**	28	32.2	26	28.3	
**Birth order**						
	**First**	37	42.5	35	38	0.480
	**Second**	25	28.7	32	34.8	
	**Third or more**	25	28.7	25	27.2	
**Family size**						
	**< 4**	7	8	7	7.6	0.620
	**4–5**	66	75.8	69	75	
	**> 5**	14	16.1	16	17.4	
**Father’s level of education**						
	**Under high school diploma**	9	10.3	43	46.8	0.001
	**High school diploma**	31	35.6	29	31.5	
	**University education**	47	54	20	21.7	
**Mother’s level of education**						
	**Under high school diploma**	22	25.2	48	52.2	0.003
	**High school diploma**	32	36.8	25	27.2	
	**University education**	33	37.9	19	20.7	
**Father’s occupation**						
	**Employee**	36	41.4	19	20.7	0.310
	**Worker**	6	6.9	24	26.1	
	**Teacher**	11	12.6	4	4.3	
	**Unemployed**	16	18.4	21	22.8	
	**Other**	18	20.7	22	26.1	
**Mother’s occupation**						
	**Employee**	9	10.3	4	4.3	0.070
	**Teacher**	16	18.4	9	9.8	
	**Housewife**	54	62.1	73	79.3	
	**Other**	8	9.2	6	6.6	

* Chi-square test

**Table 2:** Frequency distribution of the students’ bathing and combing information in the intervention and control groups.

Variables	Total number	Intervention group		Control group		P value*
		Mean	SD	Mean	SD	
**Bathing frequency**	179	2.50	1.18	2.46	1.01	0.798
**Combing frequency**	179	2.72	1.80	2.60	1.41	0.633

* T-test

Paired t-test revealed significant differences between the mean pretest and post-test scores of knowledge, constructs of the HBM, and behavior in both the intervention and control groups. As seen in Table 3, the mean scores of knowledge, perceived susceptibility, perceived severity, perceived benefits, self-efficiency, and behavior in the intervention group significantly increased one month after the intervention (P<0.05). Similar increase in scores (although to a lower extent) was observed in the control group. However, the observed differences in the control group were only significant for knowledge and perceived severity (P≤0.05). Moreover, behavior and perceived severity showed the lowest (0.57) and highest (5.3) level of change, respectively.

**Table 3:** The participants’ mean scores of knowledge, constructs of the Health Belief Model, and behaviors to prevent pediculosis.

Variable		Before the intervention		After the intervention		Mean change	P* value
		Mean	SD	Mean	SD		
**Knowledge**	**Intervention group**	12.32	2.03	14.95	1.71	2.63	< 0.001
	**Control group**	12.04	2.47	12.65	2.28	0.61	0.026
	**Independent t-test**	P = 0.414		P < 0.001			
**Perceived susceptibility**	**Intervention group**	13.86	3.31	15.68	2.98	1.82	<0.001
	**Control group**	14.25	3.67	14.31	3.22	0.06	0.845
	**Independent t-test**	P = 0.460		P = 0.004			
**Perceived severity**	**Intervention group**	10.71	3.70	16.01	3.96	5.3	<0.001
	**Control group**	12.08	3.88	12.80	3.58	0.72	0.017
	**Independent t-test**	P = 0.017		P < 0.001			
**Perceived benefits**	**Intervention group**	13.48	3.71	15.57	3.25	2.09	< 0.001
	**Control group**	13.30	4.50	13.52	4.52	0.22	0.522
	**Independent t-test**	P = 0.774		P = 0.001			
**Perceived barriers**	**Intervention group**	16.87	2.40	15.95	3.37	0.92	0.048
	**Control group**	15.43	3.43	15.16	3.72	0.27	0.592
	**Independent t-test**	P = 0.182		P = 0.007			
**Self-efficiency**	**Intervention group**	16.87	2.40	17.86	2.00	0.99	< 0.001
	**Control group**	16.40	3.07	16.76	2.54	0.36	0.260
	**Independent t-test**	P = 0.256		P = 0.002			
**Behavior**	**Intervention group**	8.41	1.70	8.98	1.29	0.57	< 0.001
	**Control group**	8.57	1.71	8.92	1.81	0.35	0.063
	**Independent t-test**	P = 0.284		P = 0.001			

* Paired t-test

Pearson’s correlation coefficient showed that behaviors to prevent pediculosis were significantly correlated with perceived barriers (P<0.001; r = 0.27) and self-efficiency (P = 0.018; r = 0.17). Knowledge was also significantly correlated with perceived susceptibility (P = 0.006; r = 0.2), perceived severity (P<0.001; r = 0.28), perceived barriers (P = 0.007; r = 0.19), perceived benefits (P = 0.005; r = 0.20), and self-efficiency (P = 0.009; r = 0.19) (Table 4).

**Table 4:** Pearson’s correlation coefficients (r) between knowledge, constructs of the Health Belief Model, and behaviors to prevent pediculosis.

Variable		Knowledge	Perceived susceptibility	Perceived severity	Perceived barriers	Perceived benefits	Self-efficiency
**Perceived susceptibility**	**R**	0.20					
	**P**	0.006					
**Perceived severity**	**R**	0.28	0.27				
	**P**	0.000	0.000				
**Perceived barriers**	**R**	0.19	0.05	-0.5			
	**P**	0.007	0.453	0.498			
**Perceived benefits**	**R**	0.20	0.28	0.221	0.11		
	**P**	0.005	<0.001	0.003	0.121		
**Self-efficiency**	**R**	0.19	0.11	0.06	0.37	0.26	
	**P**	0.009	0.122	0.397	0.000	<0.001	
**Behavior**	**R**	0.01	0.123	0.03	0.27	0.12	0.17
	**P**	0.864	0.102	0.612	<0.001	0.102	0.018

According to the first stage of regression analysis to predict behavior using the HBM constructs, only the perceived barriers were a predictor of behavior (P = 0.002) and predicted 6.9% (R^2^ = 0.069) of changes in behavior. The second stage of regression analysis, aiming to predict behavior using all the studied variables, indicated that all variables predicted 7.9% (R^2^ = 0.079) of changes in behavior (Table 5).

**Table 5:** Results of regression analysis to predict behaviors preventing pediculosis.

Variable	β	Standard error of β	t	P
**Constant**		2.916	1.054	0.294
**Combing**	0.185	0.083	2.364	0.019
**Perceived barriers**	0.234	0.043	2.823	0.005

β: Regression coefficient;R^2^ = 0.079

## Discussion

Despite advances in public health worldwide, pediculosis remains a health dilemma in undeveloped and developing countries. Head lice infestation has been reported in different parts of Iran [24]. The findings of the current study highlighted the significant effects of peer education based on the HBM on knowledge, perceived susceptibility, perceived severity, perceived benefits, perceived barriers, self-efficiency, and behaviors of students with regard to head lice infestation.

Before the intervention, the students’ knowledge about the cause of pediculosis, the age group at high risk of pediculosis, and role of head to head contact in lice transmission was considerably low. Most previous studies have reported low level of knowledge of students about this topic [6,23,25–26]. This finding is not surprising since this age group may have more important things to learn. Moreover, after the intervention, the increase in the mean score of knowledge was significantly greater in the intervention group than in the control group. These findings are in line with the results of previous studies on the effects of instruction and training on knowledge about pediculosis in students [6,23]. Gholamnia Shirvani et al. [6] and Zarban et al. [23]found that the increase in knowledge level was significantly greater in the intervention group compared to the control group. Therefore, presentation and reinforcement of simple and understandable educational content by peer education using the HBM can substantially promote the knowledge of female fifth graders about prevention of pediculosis.

Comparisons between the mean scores of behavior before and after the intervention indicated that the intervention and control groups had a significant difference in terms of the mean change in behavior scores. In fact, peer education about pediculosis effectively increased the behavior scores in the intervention group. Similar results were published by previous studies about the effects of HBM-based education on behaviors preventing parasitic or infectious diseases [18,20]. The HBM has been efficiently used to promote behaviors preventing giardiasis in students [18] and urinary infection in pregnant women [20]. Gholamnia Shirvani et al. and Zareban et al. Found improvements in the students’ performance with regard to pediculosis prevention following an educational intervention [6, 23]. The absence of significant increase in the control group’s mean score of behavior suggests that adopting behaviors to prevent pediculosis requires not only motivation and sensitization, but also an educational program about the preventive health behaviors.

The students’ mean scores of perceived susceptibility increased after the intervention. Changes in the mean scores of perceived susceptibility were significantly different in the intervention and control groups. In a review of 46 studies using the HBM, Karmel introduced perceived susceptibility as the strongest predictor of behavior [27]. Individuals who perceive themselves at risk of developing a health problem and believe that they may have a condition without experiencing its symptoms tend to avoid inappropriate behaviors leading to disease [28]. Studies on behaviors preventing urinary tract infections in pregnant women [20] and eating habits in male elementary school students [19] reported comparable results.

In the present research, the change in the mean scores of perceived severity after the intervention was significantly larger in the intervention group than in the control group. This finding can be justified by the intervention group’s enhanced knowledge about the significance of pediculosis, its complications and treatment costs following their participation in the educational program. Likewise, in a study by Ahmadi et al., 85% of the participants perceived acquired immunodeficiency syndrome as a deadly disease [29]. Moreover, Mayn Balagh et al. observed an increase in the subjects’ perceived severity after an educational intervention [19].

In the current study, the mean post-test scores of perceived benefits were higher than pretest scores in both the intervention and control groups. However, this change was significantly larger in the intervention group than in the control group. Previous studies have emphasized on the role of perceived benefits in adoption of preventive behaviors [20]. These results are in agreement with the findings of Mayn Balaghet al. about eating habits in elementary school students [19], Taghdisi and Nejhad Sadeghi about behaviors preventing urinary tract infections in pregnant women [20], and Pirzadeh and Sharifirad about the efficacy of HIV education [30].

Our findings revealed that the intervention group’s mean post-test score of perceived barriers was significantly lower than their own pretest scores and the control group’s post-test score. This difference indicates that the intervention effectively enhanced the participants’ knowledge about the barriers against adoption of preventive behaviors. Numerous studies have identified perceived barriers as the strongest predictor of preventive health behaviors [31]. Studies by Mayn Balaghet al. [19], Taghdisi and Nejhad Sadeghi [20], and Pirzadeh and Sharifirad [30] confirmed this finding.

The intervention designed in the present study significantly increased the means coreof self-efficiency. Moreover, the mean change in self-efficiency was significantly higher in the intervention group than in the control group. Mayn Balaghet al. [19] and Taghdisi and Nejhad Sadeghi [20] reported similar findings in this regard. It is well known that educational interventions need to pay utmost attention to self-efficacy as it is essential for adoption of a particular behavior [20].

The current study showed a significant correlation between the scores of perceived barriers and self-efficiency in preventive behaviors. Likewise, Mazloumi et al. reported a correlation between perceived barriers and self-efficiency in behaviors preventing diabetes [32]. Therefore, greater emphasis on perceived benefits will decrease the severity of perceived barriers. The consequent improvement in self-efficiency will then facilitate adoption of preventive behaviors. The available literature confirms this conclusion [33, 34].

There was no significant correlation between knowledge and preventive behaviors in the present study. In fact, since a variety of factors (which cannot be controlled by a particular individual, especially students) are involved in adoption of preventive behaviors, increased knowledge about high-risk populations does not entail the promotion of preventive behaviors. However, our findings revealed significant correlations between knowledge and all constructs of the HBM. Hence, promoting preventive behaviors among a specific group of people will require enhancement of their knowledge and attitude. Similarly, in studies on type II diabetes, Mazloumi et al. and Baghiani Moghadam et al. found a significant correlation between knowledge and the HBM constructs [35, 36]. The current results also showed a significant correlation between the frequency of bathing and combing and preventive behaviors such that the higher the frequency of bathing and coming, the more favorable the preventive behaviors and the health status and the lower the risk of head lice infestation. Kasiri and Rafinejad pointed to the positive efficacy of frequent showers for decreasing head lice infestation [5,15,27]. Rate of head lice infestation had an inverse correlation with level of education of parents in our study; however, Rafinejad and Safi did not find a significant association between level of education of parents and preventive behaviors [5,37].

Finally, according to regression analysis, combing and perceived barriers were identified as the only predictors of preventive behaviors. Perceived barriers were the strongest predictor of behavior. In a study conducted in Jahrom city (Iran), Azam Namdar et al. reported knowledge and perceived barriers as the predictors of behaviors preventing cervical cancer [38]. Health authorities must implement educational courses on pediculosis and provide the participants with instructions on the necessity of getting frequent haircuts, taking shower regularly, combing hair several times a day and regular change of clothes during the day.

One of the limitations of this study was the young age of participants which might have resulted in some inaccuracies in responses in the questionnaire.

## Conclusion

The findings of the current study high light the peer education based on HBM is an effective strategy to promote preventive behaviors against pediculosis among fifth grade female elementary school students in Iran.

## Supporting Information

S1 File This is the S1 File Questionnaire. (DOC)
