# Correction: Efficacy of Peer Education for Adopting Preventive Behaviors against Head Lice Infestation in Female Elementary School Students: A Randomised Controlled Trial

**DOI:** 10.1371/journal.pone.0185299

**Published:** 2017-09-19

**Authors:** Mahdi Moshki, Fereshteh Zamani-Alavijeh, Mehdi Mojadam

There is an error in affiliation 3 for the author Mehdi Mojadam. Affiliation 3 should be: Public Health Department, School of Health, Ahvaz Jundishapur University of Medical Sciences, Ahvaz, Iran.
