# Correction: Efficacy of Peer Education for Adopting Preventive Behaviors against Head Lice Infestation in Female Elementary School Students: A Randomised Controlled Trial

**DOI:** 10.1371/journal.pone.0212625

**Published:** 2019-02-19

**Authors:** Mahdi Moshki, Fereshteh Zamani-Alavijeh, Mehdi Mojadam

There is some overlap between work reported in this article [1] and a previous publication by the same research group [2] that is not cited or discussed in the *PLOS ONE* article. The *Journal of Health and Development* article [2] presents the development and validation of the questionnaire which involved a cross-sectional approach, and the *PLOS ONE* article [1] reports a randomized interventional study that used this tool. While the overall aims of the articles differ, the development and validation of the questionnaire are reported in both articles. Compared to the earlier article, the *PLOS ONE* article provides additional validation information including the following: detailed question items, scoring protocols, and an explanation of the interpretation method.

Both articles report use of the same study sites, sampling method, and number of participants in describing validation and intervention aspects of this work. The authors confirm that they used an independent cohort of participants for questionnaire development and validation [1,2]; the participants for these phases of the work resided in the same city but were at different schools. However, the study reported in *PLOS ONE* uses the same participants as the earlier study reported previously.

The authors apologize for not citing the earlier article in the *PLOS ONE* publication.

## Supporting information

S1 File Flowchart. This flowchart illustrates the relationship and overlap between the *PLOS ONE* article and the *Journal of Health and Development* article.
(PDF)
