# Supplementary material for: Efficacy of Peer Education for Adopting Preventive Behaviors against Head Lice Infestation in Female Elementary School Students: A Randomised Controlled Trial
Source: PLoS One. 2017 Jan 10;12(1):e0169361. doi: 10.1371/journal.pone.0169361 (PMC5224824; doi:10.1371/journal.pone.0169361)
Supplement: S1 File — (DOC) [file pone.0169361.s001.doc]

**Head Lice Infestation Questionnaire**

**Student’s Demographic Characteristics**

**Age:**

**Are you the oldest, the youngest or the middle child?**

**How many siblings do you have?**

**What is your father's occupation?**

Employee Worker Teacher Unemployed Other

**What is your mother's occupation?**

Employee Worker Teacher Unemployed Other

**What is your father's level of education?**

Under high school diploma High school diploma University education

**What is your mother's level of education?**

Under high school diploma High school diploma University education

**How many times a week do you take a bath?**

**How many times a day do you comb your hair?**

**Whether you've been infected with head lice in the past 3 months?** Yes No

**Had any of your family member infected with head lice in the past 3 months?** Yes No

**Have you received training in the field of head lice infestation in the last 3 months?** Yes No

**Health Belief Model Questionnaire**

|  | **Knowledge questions** | **Yes** | **No** | **I do not know** |
| --- | --- | --- | --- | --- |
|  | **Head lice are more common in long hair.** |  |  |  |
|  | **Head lice can survive for several days away from the human host.** |  |  |  |
|  | **Person with head lice is permanently scratching his head.** |  |  |  |
|  | **Head lice spread from person to person when people's heads touch or after sharing things like hats and other clothing, combs, brushes, headbands, or barrettes.** |  |  |  |
|  | **Head lice spread from person  to person** |  |  |  |
|  | **Small white eggs or nits (egg cases) of head lice can be spotted in the hair behind the ears or at back of the neck.** |  |  |  |
|  | **If head lice are found in the family, the family home must be cleaned thoroughly.** |  |  |  |
|  | **Head lice can be spread by pets and pet birds.** |  |  |  |
|  | **For the treatment of head lice, lice shampoo should be used twice.** |  |  |  |

|  | **Perceived Susceptibility questions** | **I quite agree** | **I agree** | **I have no idea** | **I disagree** | **I quite disagree** |
| --- | --- | --- | --- | --- | --- | --- |
|  | **Where I live or study, the risk of head lice infestation is high.** |  |  |  |  |  |
|  | **Head lice, mostly infect elementary school students.** |  |  |  |  |  |
|  | **There is likely to get head lice at any age.** |  |  |  |  |  |
|  | **I'm worried about getting infected with head lice myself.** |  |  |  |  |  |
|  | **I'm worried about my family members to be infected by head lice.** |  |  |  |  |  |

|  | **Perceived Severity questions** | **I quite agree** | **I agree** | **I have no idea** | **I disagree** | **I quite disagree** |
| --- | --- | --- | --- | --- | --- | --- |
|  | **Head lice infestation causes severe itching in infected person.** |  |  |  |  |  |
|  | **Head lice infestation make students restlessness and anxious.** |  |  |  |  |  |
|  | **Because of pediculosis capitis, I cannot pay attention to the teacher.** |  |  |  |  |  |
|  | **Head lice do not let me to learn the lessons.** |  |  |  |  |  |
|  | **If I get head lice, my friends will not play with me.** |  |  |  |  |  |

|  | **Perceived Barriers questions** | **I quite agree** | **agree** | **No idea** | **I disagree** | **Quite disagree** |
| --- | --- | --- | --- | --- | --- | --- |
|  | **Combing several times a day is hard for me.** |  |  |  |  |  |
|  | **I forget to use my own towels and comb.** |  |  |  |  |  |
|  | **Using anti-lice shampoo causes my scalp irritation.** |  |  |  |  |  |
|  | **Bathing is a disaster for me.** |  |  |  |  |  |
|  | **When I'm in a hurry to go to school in the morning, I have no time for combing my hair.** |  |  |  |  |  |

|  | **Perceived Benefits Questions** | **I quite agree** | **agree** | **I have no idea** | **I disagree** | **I quite disagree** |
| --- | --- | --- | --- | --- | --- | --- |
|  | **Combing the hair on a regular basis, several times a day, is effective in preventing head lice infestation.** |  |  |  |  |  |
|  | **Timely treatment of head lice infestation can prevent the infection of others.** |  |  |  |  |  |
|  | **Head lice prevention is cheaper than its treatment.** |  |  |  |  |  |
|  | **Through head lice infestation Ican have more friends.** |  |  |  |  |  |
|  | **I look better if I do not have head lice.** |  |  |  |  |  |

|  | **Self-Efficacy questions** | **I quite agree** | **I agree** | **I have no idea** | **I disagree** | **I quite disagree** |
| --- | --- | --- | --- | --- | --- | --- |
|  | **If I scheduled comb my hair several times a day, I can do it.** |  |  |  |  |  |
|  | **When I decide to comb my hair regularly, my focus will be strictly on it.** |  |  |  |  |  |
|  | **I use my personal belongings (such as scissors, combs and towels) when I go to the hairdresser.** |  |  |  |  |  |
|  | **If I suddenly get infected with head lice, I can not handle it.** |  |  |  |  |  |
|  | **One of my personal characteristics is that I do not like bathing.** |  |  |  |  |  |

|  | **Cues to action questions** |
| --- | --- |
|  | **Which of the following would be most helpful for preventing head lice infestation?**  **Teacher Parents Doctor Classmate and Friends TV programs such as cartoon and health programs**  **My own information Fear of getting head lice** |
|  | **Which of the following do you consider acceptable in head lice infestation prevention?**  **Teacher Parents Doctor Classmate and Friends TV programs such as cartoon and health programs**  **My own information Fear of getting head lice** |

|  | **Behavioral questions** | **Yes-always** | **Yes-sometimes** | **No- never** |
| --- | --- | --- | --- | --- |
|  | **In the past month, I've combed my hair several times a day.** |  |  |  |
|  | **In the past month, I've gone to the bathroom regularly.** |  |  |  |
|  | **In the past month, I've cleaned my brush and comb every day.** |  |  |  |
|  | **In the past month, I've used my personal belongings (such as combs, brushes and scissors) at the hairdressers.** |  |  |  |
|  | **In the past month, I've used my own personal items (such as scarves, bed linens, combs and brushes) at home.** |  |  |  |
