# Supplementary material for: Correction: Efficacy of Peer Education for Adopting Preventive Behaviors against Head Lice Infestation in Female Elementary School Students: A Randomised Controlled Trial
Source: PLoS One. 2019 Feb 19;14(2):e0212625. doi: 10.1371/journal.pone.0212625 (PMC6380576; doi:10.1371/journal.pone.0212625)
Supplement: S1 File — This flowchart illustrates the relationship and overlap between the PLOS ONE article and the Journal of Health and Development article. (PDF) [file pone.0212625.s001.pdf]

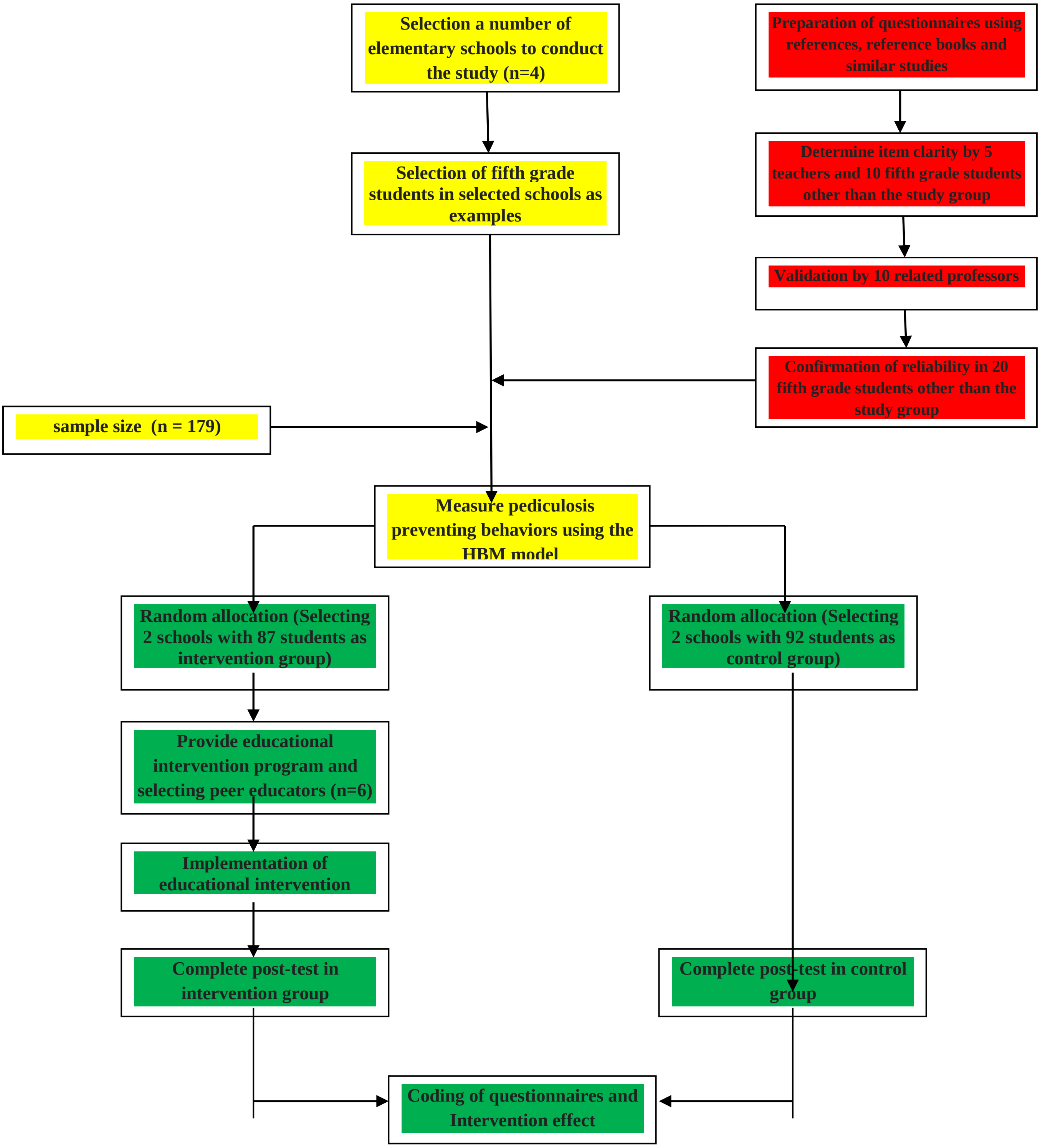


Yellow color indicating section published in Health and Development journal.

Green color indicating section published in PLOS one

Red color is a partial duplicate section in both journals which we have already explained. Tools development and standardization has been explained in more details in PLOS one. Also we have provided the questionnaire as supporting information which has not been publish before.
